# Synthesis, Physical Properties and Application of a Series of New Polyoxometalate-Based Ionic Liquids

**DOI:** 10.3390/molecules26020496

**Published:** 2021-01-18

**Authors:** Yohan Martinetto, Salomé Basset, Bruce Pégot, Catherine Roch-Marchal, Franck Camerel, Jelena Jeftic, Betty Cottyn-Boitte, Emmanuel Magnier, Sébastien Floquet

**Affiliations:** 1 Institut Lavoisier de Versailles, UMR 8180 CNRS, Université de Versailles St-Quentin en Yvelines, Université Paris-Saclay, 78035 Versailles, France; martinetto.yohan@hotmail.fr (Y.M.); salome.a.basset@gmail.com (S.B.); bruce.pegot@uvsq.fr (B.P.); catherine.roch@uvsq.fr (C.R.-M.); emmanuel.magnier@uvsq.fr (E.M.); 2 Institut des Sciences Chimiques de Rennes, UMR 6226, Université de Rennes 1, 35042 Rennes, France; jelena.jeftic@ensc-rennes.fr; 3 Institut Jean-Pierre Bourgin, INRAE, Agro Paris Tech, Université Paris Saclay, 78000 Versailles, France; betty.cottyn@inra.fr

**Keywords:** polyoxometalate, ionic liquid, viscosity, rheology, catalysis, oxidation, alcohol

## Abstract

This paper deals with the preparation and the characterization of four new ionic liquids resulting from the pairing of various polyoxotungstates or polyoxomolybdates with the cation trihexyltetradecylphosphonium. The physical properties measured by different techniques evidence that the viscosity and the rheological behaviors of such POM-based ionic liquids, POM-ILs, strongly depend on the nature of the POM, especially its charge. Playing on the nature of the POM, we can indeed obtain Newtonian liquids or some much more viscous materials exhibiting characteristics of resins or pseudo-plastics. In a second part of this study, the potentialities of using such materials both as solvent and catalyst for the oxidation of a series of alcohols are presented as proof of concept. This part highlights great differences in strength and selectivity as a function of the POM-IL used. Furthermore, a very simple way to recycle the catalyst is also presented.

## 1. Introduction

Polyoxometalate (POM) compounds constitute a very wide class of inorganic molecules, for which the composition, the size and the charge can be precisely adjusted. Owing to their structural and compositional versatility, much of the current research in POMs chemistry is driven by potential applications in numerous fields such as in supramolecular chemistry [1,2,3,4], nanotechnology [5,6], medicine [7,8,9,10,11], magnetism [12,13,14], catalysis [15,16,17,18,19,20,21,22] or electrocatalysis [23,24,25,26], which explains that the number of publications concerning polyoxometalates has exploded over the last two decades.

The choice of the constituents drives the final architectures and their physical properties [27,28,29,30,31,32,33]. The elaboration of hybrid functional POM-based molecules by grafting organic groups or coordination complexes offers a large panel of possibilities allowing to fine-tune their solubility, their charge, their electronic properties, as well as their capabilities to self-organize onto various surfaces [34,35] and to interact with biomolecules, membranes, or proteins. Besides, such assemblies permit to combine the properties of the POM with the properties of the appended subunit for the design of multifunctional devices [32,36,37].

Often mistakenly considered as spectator ions, the counter-cations can also play a fundamental role in the design of functional hybrid POM-based materials [38]. In particular, as emphasized by Wu in a recent review, the electrostatic coupling of POMs with more or less sophisticated organic cations leads to a rich family of hybrid compounds at the boundary between molecular chemistry and chemistry of materials [39]. For instance, the encapsulation of POMs within tetraalkylammonium cations featuring one or two long hydrocarbon chains leads to the so-called Surfactant Encapsulated Clusters (SEC), which can be used to prepare gels, Langmuir-Blodgett films [40,41], modified electrodes [42] or materials for catalysis [43]. Among these materials, the choice of the organic cations can also lead to the formation of ionic liquid crystals [44,45,46] if the material can be ordered or ionic liquids in the other case. These two domains are relatively new since the first publications appeared in 2004–2005 and the case of ionic liquids built from pairing POMs and organic cations is undoubtedly not clearly understood. To be called an ionic liquid, a salt must be an isotropic liquid and must have a melting point below 100 °C. In the literature, it is interesting to note that the combination of the two keywords “polyoxometalate” and “ionic liquids” (IL) entered in the Web of Knowledge gives almost 600 hits. However, a careful investigation of these papers demonstrates an abuse of language in at least 90% of them. In fact, we identified only a few dozen publications which report real POM-based ionic liquids which are called POM-ILs hereafter. Furthermore, their physical characterizations are often limited to the determination of the melting point and visual observation or with a microscope of a liquid state above this temperature. Consequently, we can consider that this domain remains relatively poorly explored, despite a likely great interest of such systems for many applications. As example, we can cite the common work of Streb and Mitchell, who demonstrated the potentiality of POM-ILs in the preservation of historical heritage, depollution or as anti-corrosion agent for metals [47,48,49,50,51]. We can also mention very few works using POM-ILs for catalytic oxidation [52], while more studies are focused on POMs solubilized in classical ILs or on the use of ionic liquids based of peroxomolybdates and peroxotungstates species [53,54].

Considering the lack of data on POM-ILs and their possibilities to be used as solvent and catalyst in the oxidation of various organic substrates, several objectives are pursued in the present study.

**Figure 1:**
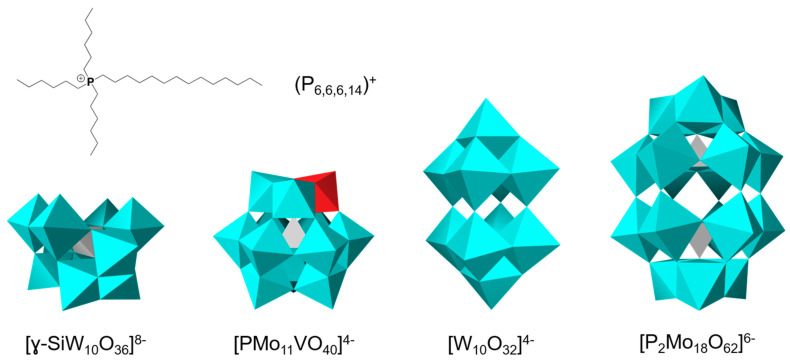
Representation of the cation and the POMs used in this study. MO_6_ octahedra (M = Mo or W) are given in blue, grey tetrahedra correspond to SiO_4_ or PO_4_ central tetrahedra and VO_6_ octahedron in the POM [PMo_11_VO_40_]^4−^ is highlighted in red.

First, this study aims to investigate and compare the properties of ionic liquids formed by combining trihexyltetradecylphosphonium cations with a series of four POMs, which differ in their size, their composition, and their charge (see Figure 1). In this part, we will particularly focus our attention on the rheological properties of such systems in comparison with the starting ionic liquid salt P_6,6,6,14_Cl and with the compound (P_6,6,6,14_)_4_[W_10_O_32_] which displays all the properties required to act as a good solvent for organic molecules (low melting point below −10 °C, dielectric constant of about 3) [55]. These data are barely investigated in the literature and we aim to evidence that changing the POM can dramatically modify the physical properties of the ionic liquid, a key point for further applications. Moreover, acquiring more and more data on physical properties of POM-ILs will permit a better understanding of the expected behavior of POM-ILs and will allow to elaborate predictive models in our future studies.

In a second part, we strive to demonstrate that these systems can be used both as solvent and catalyst in the oxidation of organic molecules. Such a system can also address several issues encountered in homogeneous catalysis, notably in terms of environmental impacts due to the use of organic solvents, of reaction mixture separation and of recyclability of the catalyst. For this purpose, a weakly viscous POM-IL at relatively low temperature is required and the choice of the system is of prime importance.

## 2. Results and Discussion

### 2.1. Syntheses and Characterizations of POM-Based Ionic Liquids

#### 2.1.1. Syntheses and Routine Characterizations

To design new POM-ILs, the choice of cations and POMs are of great importance. In this study, we selected four POMs considered to be representative of the diversity of these anionic species: two tungstates and two molybdates ones. Among them, one is an isopolyoxometalate and three others are heteropolyoxometalates including one vacant POMs, one 3d metal substituted Keggin-type POM and one Dawson-type POM. All of them are reported to be catalysts in oxidation processes in agreement with one of our objectives. Finally, this series also displays charges ranging from 4^−^ to 8^−^, a key-parameter for the pairing with cations.

As evidenced in our recent review, tetraalkylammonium and tetraalkylphosphonium bearing four long alkyl chains are good candidates to get POM-ILs [46]. As shown by Yan and coworkers [56,57,58] when comparing the melting points of ((*n*-C_10_H_21_)(*n*-C_4_H_9_)_3_N^+^)_5_[SiW_11_VO_40_] (+95 °C) and ((*n*-C_14_H_27_)(*n*-C_4_H_9_)_3_P^+^)_5_[SiW_11_VO_40_] (+50 °C), the choice of phosphonium cations instead of ammonium favors the decrease of the fusion temperature. In addition, the pioneering work of Rickert [59,60] and the more recent study of Nogueira [61] evidence that the trihexyltetradecylphosphonium cation leads to POM-ILs displaying melting points below room temperature. This is a required parameter to use such materials as solvent in oxidation reactions with soft conditions. We decided to focus our attention on this cation. Besides, to our knowledge, such POM-ILs have never been tested for catalytic applications.

Two methods were considered to prepare the compounds **1**–**4**, as shown in the experimental section. The compound **1**, namely (P_6,6,6,14_)_4_[W_10_O_32_] was obtained by direct synthesis of the POM as described in the literature (see Materials and Methods) followed by the separation of the ionic liquid phase after the addition of the P_6,6,6,14_Cl salt. In contrast, for compounds **2**–**4**, the POM precursors, namely [SiW_10_O_36_]^8−^, [PMo_11_VO_40_]^4−^ and [P_2_Mo_18_O_62_]^6−^ were prepared as acid, potassium or sodium salts as described in the literature before cationic metathesis in presence of an excess of P_6,6,6,14_Cl in a water/ethanol mixture. The reaction mixtures are stirred during 24 h to reach the completeness of the cationic exchange and rule out the formation of mixed POM-IL phases including phosphonium and alkali cations. Indeed, in biphasic mixtures (ionic liquid phase and water/ethanol phase), protons or alkali cations prefers to pass within the phase rich in water while the quaternary phoshonium cations are poorly soluble in such medium and highly soluble in ionic liquid phase.

After drying under vacuum, compounds **1**–**4** were isolated as colourless or pale-yellow oils. The four compounds were characterized by FT-IR, NMR and TGA. The FT-IR spectra were recorded at room temperature (Figures S1–S4, ESI) on an IR spectrometer equipped with an ATR diamond apparatus. In the four cases, the FT-IR spectra display the vibration modes of the organic cations associated with the expected POM, which indicates that the integrity of the POM during the experimental procedure is maintained. Not only used for qualitative characterization of **1**–**4**, infrared spectroscopy represents also a powerful tool to investigate the organization and dynamics of the alkyl chains of the cations associated to the POM [45,62,63,64,65]. In the high frequency region, the two weak bands observed at about 2953–2955 cm^−1^ and 2870–2871 cm^−1^ can be assigned to the antisymmetric (ν_as_(CH_3_)) and symmetric (ν_s_(CH_3_)) stretching vibrations of the terminal methyl groups, while the two strong bands found at around 2919–2926 cm^−1^ and 2850–2855 cm^−1^ are assigned to the antisymmetric (ν_as_(CH_2_)) and symmetric (ν_s_(CH_2_)) stretching vibrations of the methylene groups. These two bands are very useful to study the order of the alkyl chains in the ionic materials. Indeed, low frequencies (2915–2918 and 2846–2850 cm^−1^) are indicative of a highly ordered chain, in ionic liquid crystals for instance, while their blue shift towards 2924–2928 cm^−1^ and 2854–2856 cm^−1^ indicates a larger conformational disorder. In compounds **1**–**4**, these bands are found in the 2924–2926 and 2854–2855 wavenumber ranges, typical of disordered alkyl chains expected in isotropic liquids.

**Figure 2:**
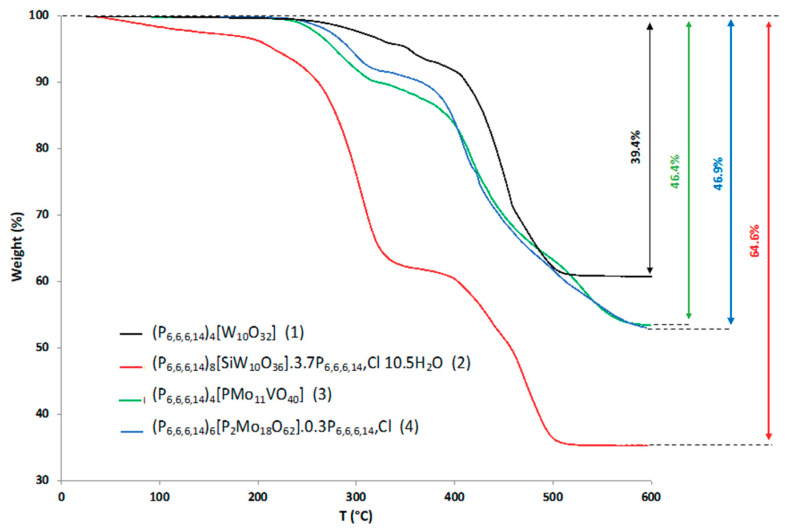
TGA curves recorded for compounds **1**–**4** under O_2_ flow; heating rate of 5 °C/min.

Thermogravimetric analyses performed under O_2_ flow in the 20–600 °C temperature range allows establishing a complete chemical formula for **1**–**4**. As shown in Figure 2, the TGA traces show that compounds **1**, **3** and **4** are anhydrous since no removal of water is detected before 200 °C. In contrast, for **2**, about 10 water solvate molecules are detected. The decomposition temperatures are found higher than 220 °C for **1**, **3** and **4** and around 190 °C for compound **2**, which demonstrates a thermal stability high enough to envision application in catalysis under heating. Finally, the decomposition of the phosphonium cations and the excess of chloride in some cases is observed up to 600 °C. This step is the most informative one for such ionic systems. It allows establishing the chemical formulae for each compound: (P_6,6,6,14_)_4_[W_10_O_32_] for **1**; (P_6,6,6,14_)_8_[SiW_10_O_36_]·3.7P_6,6,6,14_Cl 10.5H_2_O for **2**; (P_6,6,6,14_)_4_[PMo_11_VO_40_] for **3** and (P_6,6,6,14_)_6_[P_2_Mo_18_O_62_]·0.3P_6,6,6,14_Cl for **4**. For all compounds, the TGA curves indicates either the expected stoichiometry or an excess of phosphonium cations per POM in agreement with the complete cationic exchange during the synthesis. It is interesting to note that when the charge increases, we observe a tendency of the POM to interact with more cations. It was not reported so far with alkylammonium salts or with phosphonium-based POM-ILs for which the examples reported in the literature include POM with charges ranging only from 2^−^ to 5^−^ [56,57,58,59,60,61]. This phenomenon is particularly important with [SiW_10_O_36_]^8−^ and was also observed for [PW_11_O_39_]^7−^ and [P_8_W_48_O_184_]^40−^ (unpublished results). Despite our efforts, we were not able to remove the excess of phosphonium chloride, which suggest that all of the cations strongly interact with the POM.

**Figure 3:**
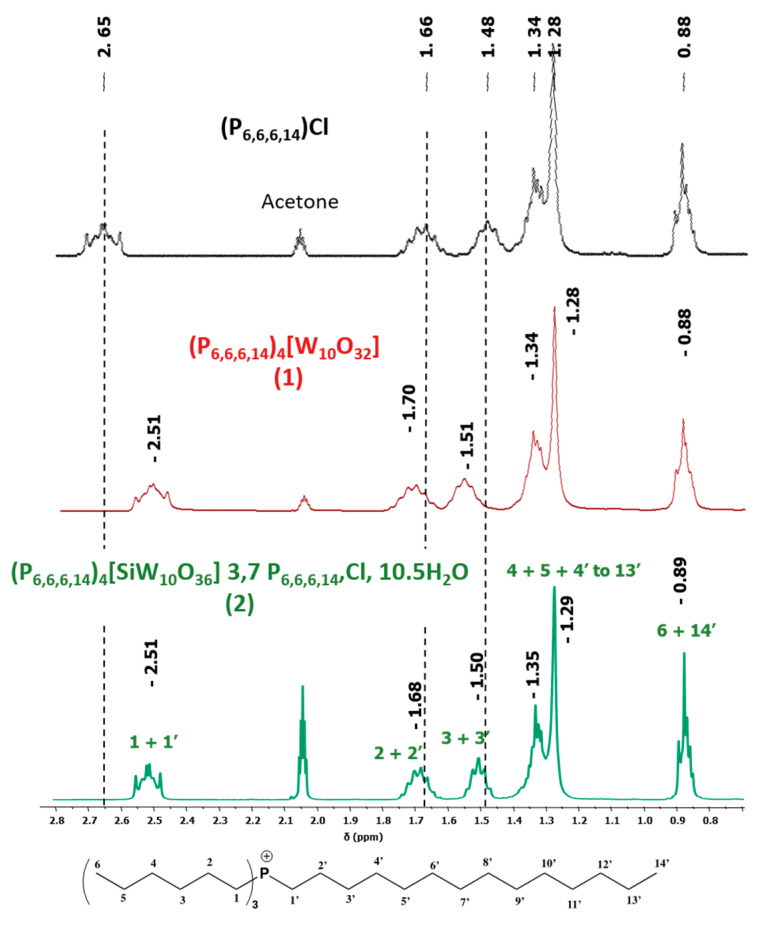
^1^H NMR spectra comparing (P_6,6,6,14_)_4_[W_10_O_32_] (**1**), (P_6,6,6,14_)_8_[SiW_10_O_36_]·3.7P_6,6,6,14_Cl 10.5H_2_O (**2**) and (P_6,6,6,14_)Cl in acetone-*d*_6_.

^183^W, ^29^Si and ^31^P solution NMR spectra of **1**–**4** confirm the presence, the nature and the purity of the expected POMs within the isolated viscous liquids (Figures S5–S8, ESI). ^1^H-NMR spectra allow characterizing the cations within the materials. As seen in Figure 3 and in Figures S9 and S10 (ESI), as a general feature for compounds **1**–**4**, the ^1^H-NMR spectrum of the trihexyltetradecylphosphonium cations indicates significant shifts in both directions of the methylenic protons when associated to the POM. On the other hand, the linewidth of the signals appears weakly or not affected by the association, in contrast with previous results obtained with POM-based ionic liquid crystals [45]. This suggests that the POM/cation interaction is probably weaker when tetraalkylammonium or phosphonium cations with long alkyl chains are used compared to highly dissymmetrical cations used for the design of liquid crystals. Interestingly, as depicted in Figure 3, while the formula of **2**, namely (P_6,6,6,14_)_8_[SiW_10_O_36_]·3.7P_6,6,6,14_Cl 10.5H_2_O, gives a significant excess of phosphonium cations, the ^1^H-NMR spectrum evidences only one set of signals for the protons of the phosphonium cations with shielding and deshielding effects similar to that observed in **1**, **3** and **4**. It means that either the interacting and free cations are undergoing a fast exchange or that all the cations interact similarly with the POM due to its higher charge and thus a stronger electrostatic attraction force. This hypothesis suggests that when the charge of the POM increases, the number of cations interacting with the surface of the POM is higher than the stoichiometry expected and therefore that the number of counter cations is governed by the surface of the POM. The electroneutrality is then assured by chloride anions. This must be clarified by DFT calculation.

#### 2.1.2. Determination of Physical Properties

##### Differential Scanning Calorimetry and Polarized Optical Microscopy

**Figure 4:**
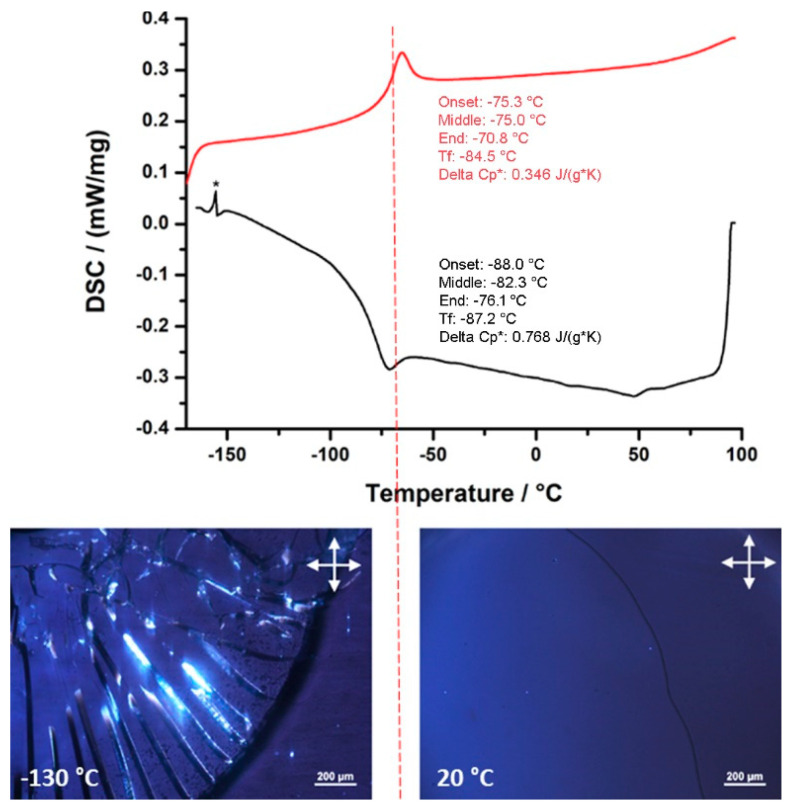
DSC traces in the heating (red) and the cooling (black) modes for compound **2**. Images taken at −130 °C and +20 °C by Polarized Optical Microscopy (* = artefact).

Differential Scanning Calorimetry (DSC) was performed in the −150 °C to +100 °C temperature range under nitrogen atmosphere on compounds **1**–**4** (see Figure 4 and Figure S11, ESI). In contrast with phase transitions observed in liquid crystals [45], the glass transitions are more difficult to observe because the enthalpy variation due to the melting of the solid is expected to be zero. Nevertheless, we observe a variation of C_p_ and a small variation of enthalpy due to the reorganization of the alkyl chains, which leads to a significant variation of the DSC curves as shown in Figure 4 and in Figure S11 in the ESI for **1**–**4**. These experiments give a significant modification on the heating curves of each compound which is unambiguously attributed to glass transitions thanks to polarized optical microscopy experiments performed below and above these transitions (Figure 4 (bottom), Figure S12, ESI). For instance, in Figure 4, the pictures are taken at −130 °C and +20 °C while the melting point is identified around −70 °C. Neither of the pictures exhibit any birefringence which could be observed in the case of an ordered phase. The texture at −130 °C shows a brittle solid in an isotropic glassy state, while the compound is a viscous, isotropic and malleable material at +20 °C. The conclusions are the same for P_6,6,6,14_Cl and **1**, **3** and **4**. The glass transition temperatures are gathered in Table 1.

##### Rheological Investigations

To demonstrate the ionic liquid nature of our compounds and determine their physical properties, the rheological behavior of all the compounds was investigated with a Haake MARS III controlled-stress rheometer equipped with a cone-plate geometry (diameter = 35 mm, angle = 1°) and a Peltier thermal regulator. The main conclusions are summarized in Table 1.

The commercial salt P_6,6,6,14_Cl appears as a colorless fluid at room temperature. The evolution of the shear stress (τ) as a function of the shear rate ($\dot{\gamma }$) at various temperatures is given in Figure S13a (ESI). From the observed straight line flow curves, it appears that the compound is a Newtonian fluid (τ = η × $\dot{\gamma }$, where η is the viscosity) in the 20–100 °C temperature range and that the viscosity (i.e., the slope of the curves) gradually decreases with the increase of the temperature. Figure S13b presents the evolution of the viscosity as a function of the temperature. At room temperature, the viscosity of P_6,6,6,14_Cl is around 0.86 Pa.s and is close to that of ricin oil (0.98 Pa.s). Upon temperature increase, the viscosity gradually decreases to reach 0.04 Pa.s at 100 °C. The loss modulus (G″) and the storage modulus (G′) were also measured as a function of the temperature between 20 and 100 °C (Figure S13c, ESI). Generally, G′ concerns elastic properties of the material, while G″ represents the viscous nature of the compound. The G″ modulus is higher than G′ at room temperature, meaning that this compound shows a rather viscous character. The G″ modulus decreases with the temperature to reach low values at high temperature at the level of the storage modulus values. These results confirm that P_6,6,6,14_Cl compound is a slightly viscous ionic liquid.

**Figure 5:**
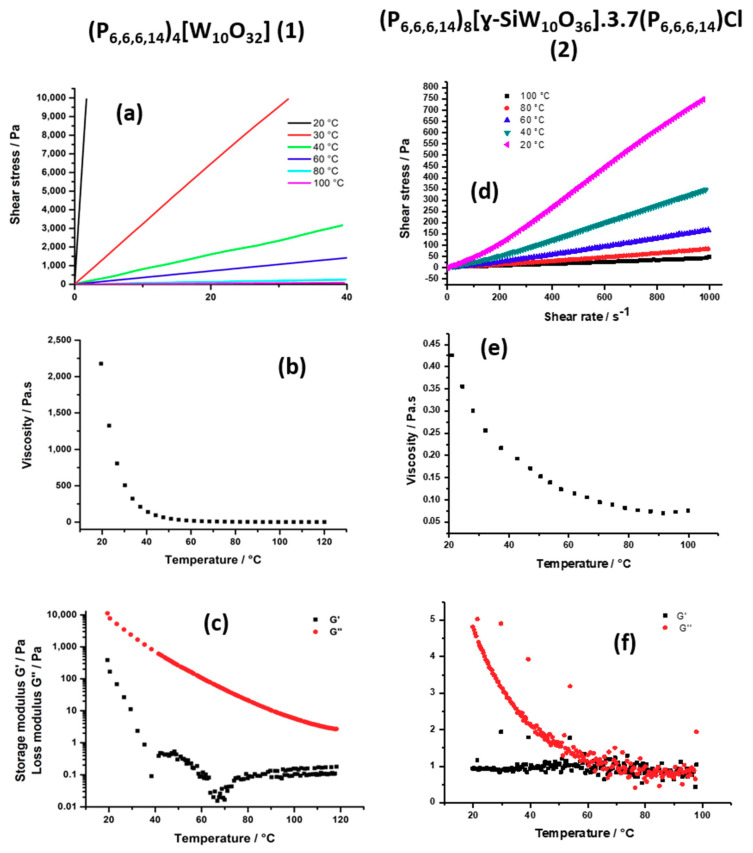
Flow curves τ = f($\dot{\gamma }$) measured between 20 and 100 °C for **1** (**a**) and for **2** (**d**); Viscosity variation as a function of the temperature between 20 °C and 100 °C ($\dot{\gamma }$ = 10 s^−1^) for **1** (**b**) and **2** (**e**); Temperature dependence of G′ (black dots) and G″ (red dots) with 20% strain and f = 1 Hz for **1** (**c**), and **2** (**f**).

Substituting the chloride anions by the [W_10_O_32_]^4−^ anion (compound **1**) leads to the increase of the viscosity from 0.86 to 2200 Pa.s^−1^ but **1** is still a Newtonian liquid at room temperature, as confirmed by the linear dependence observed between the shear stress (τ) and the shear rate ($\dot{\gamma }$) (Figure 5a). The viscosity drastically decreases with the temperature increase. The viscosity is below 100 Pa.s above 40 °C and reaches 0.92 Pa.s at 100 °C, which is the viscosity of P_66614_Cl at room temperature (Figure 5b). The loss modulus (G″) and the storage modulus (G′) were measured as a function of the temperature between 20 and 100 °C (Figure 5c). Both modulus values decrease with temperature increase. G″ is at least two orders of magnitude larger than G′ over the whole temperature range explored, meaning that this compound has a mainly viscous behavior. These results demonstrate that this compound is a highly viscous room temperature ionic liquid (RTIL) which becomes more and more fluid as the temperature increases.

**Figure 6:**
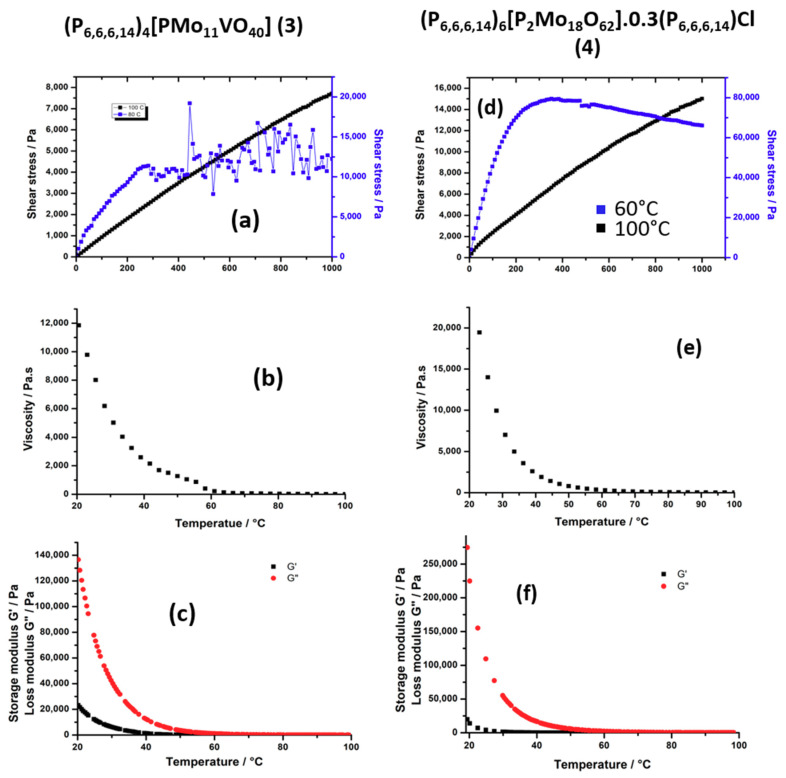
Flow curves τ = f($\dot{\gamma }$) measured at 80 and 100 °C for **3** (**a**) an 60 and 100 °C for **4** (**d**); Viscosity variation as a function of the temperature between 20 °C and 100 °C ($\dot{\gamma }$ = 10 s^−1^) for **3** (**b**) and **4** (**e**); Temperature dependence of G′ (black dots) and G″ (red dots) with 20% strain and f = 1 Hz for **3** (**c**) and **4** (**f**).

Compound **3**, (P_6,6,6,14_)_4_[PMo_11_VO_40_], is a highly sticky paste at room temperature. The rheological measurements confirm that this compound is a highly viscous fluid at room temperature (G″ >> G′) (Figure 6c) and shows that the viscosity decreases drastically between 20 °C and 100 °C (Figure 6b). The evolution of the shear stress (τ) as a function of the shear rate ($\dot{\gamma }$) was found to be very difficult to measure below 80 °C. Above 80 °C, the curves show that the shear stress strongly decreases when the shear rate increases (Figure 6a). This effect is much more marked around 80 °C. This behavior is typical of a shear thinning non-Newtonian fluid which viscosity decreases under shear strain (pseudoplastic behavior). At room temperature, the viscosity is very high (~12,000 Pa.s) and thus, this compound can be categorized as a resin-like material. Compared to (P_6,6,6,14_)_4_[W_10_O_32_] with an anion of the same charge, these results clearly show that a slight increase of size of the POM has a strong effect on the rheological behavior. This is in agreement with our previous works on ionic liquid crystals built with bigger POMs, i.e., a derivative of the cyclic POM “P_8_W_48_” [45] and different types of giant spherical Keplerates “Mo_132_” [44,66,67]. In these cases, the size of the POM fills in the range 2–3 nm, while the anionic charge varies in the 32^−^ to 54^−^ range. In each case we got a highly viscous paste, which never became fluid even in the liquid crystal phase.

Compound **4**, (P_6,6,6,14_)_6_[P_2_Mo_18_O_62_]·0.3P_66614_Cl, behaves like **3**. This highly viscous compound displays a pseudoplastic behavior below 100 °C and the viscosity clearly decreases with the shear rate (Figure 6e). This compound can also be categorized as a resin. The viscosity measured at 100 °C is close to that of honey (10–20 Pa.s). In this compound, both the size and the charge of the POM were increased, and this leads to a further increase of the viscosity by 160% compared to (P_6,6,6,14_)_4_[PMo_11_VO_40_], despite the presence of some additional P_6,6,6,14_Cl per unit.

Finally, the presence of almost four additional P_6,6,6,14_Cl salts in the formula of **2** ((P_6,6,6,14_)_8_[SiW_10_O_36_]·3.7P_6,6,6,14_Cl 10.5H_2_O) also seems to have a strong influence on the rheological behavior of this hybrid POM. Compound **2**, which has a more charged anion (8^−^) but a size close to that of [W_10_O_32_]^4−^, was isolated with 3.7 additional P_6,6,6,14_Cl per unit. This compound is now a liquid at room temperature with a viscosity even lower than that of (P_6,6,6,14_)_4_[W_10_O_32_]. The viscosity is close to that of honey at room temperature and decreases with the temperature increase to reach a viscosity at 100 °C close to that of ricin oil (Figure 5e). The flow curves show that this compound displays a weak shear thickening behavior at low temperatures (Figure 5e) (the shear stress slightly increases when the shear rate increases) and thus can be classified as a dilatant. The measurements of the loss modulus (G″) and the storage modulus (G′) confirmed that this compound has mainly a viscous character (G″ >> G′) (Figure 5f). Unfortunately, due to the presence of free P_6,6,6,14_Cl, the exact impact of the charge on the rheological behavior could not be properly evaluated. The number of free P_6,6,6,14_Cl seems to depend on the charge of the anion. It can be noticed that, in all cases, the measured viscosity was always higher compared to that of the pure P_6,6,6,14_Cl.

In summary, the rheological measurements demonstrated that the rheological behavior of POM hybrids strongly depends on the charge and the size of polyoxometalates as well as on the number of extra (P_6,6,6,14_Cl) associated. This number seems also to depend on the charge of the POM and the strength of the electrostatic interaction between POM and cations. An increase of the size clearly leads to a drastic increase of the viscosity and can lead to the isolation of pseudoplastic resin. In the present study, the effect of the charge is not so easy to evaluate, since the increase of the charge is also associated to the increase of the number of additional salt (P_6,6,6,14_Cl). So, the effect of the charge on the viscosity can be counterbalanced or enhanced by the presence of P_6,6,6,14_Cl. Nevertheless, the formation of room-temperature ionic liquid from fluid cationic surfactants should be favored using small sized and weakly charged POM. The isolation of ionic liquids with highly charged POMs can be favored by incorporating additional free surfactants (dilution with the fluid cationic surfactants).

### 2.2. Application of POM-ILs in Catalysis

Oxidation of alcohols using catalytic systems based on transition metal and heavy metal ions is a very important organic synthesis pathway to the corresponding carboxylic acids [68,69]. POMs have been widely used for that purpose and revealed to be very active in presence of H_2_O_2_ which is the most environmentally friendly oxidant after molecular oxygen [70,71]. The use of POMs dissolved in “classical” ionic liquids have a growing interest and have been successfully used in alcohol transformation to aldehydes and less frequently to acids [46], but surprisingly, real POM-ILs that are liquid below 100 °C are not used so often in catalytic reactions. To our knowledge, we found only one work by Qiao in 2009 on this topic [52].

In the present study, we evidenced above that small changes in the nature of the POM can dramatically modify the physical properties of the materials. Our goal is also to see if these changes could also have significant effect on catalysis. At 100 °C, as seen in Table 1, the viscosity values are small enough to be used as relatively fluid solvent. Besides, compounds **1**–**4** are also capable of dissolving small organic molecules. We decided to compare the four POM-ILs as solvent and catalyst in the oxidation reaction of various alcohols in presence of H_2_O_2_ containing an aqueous phase as co-oxidant.

To evaluate the efficiency of the 4 POM-ILs of this study in catalysis, they were first tested in the oxidation of 2-trifluorobenzyl alcohol. The experiments were performed accordingly to the protocol described in the experimental section, in a biphasic system (POM-IL phase–aqueous phase containing H_2_O_2_ as cocatalyst) at 90 °C to avoid evaporation of the aqueous phase and to be fluid enough to stir efficiently the reaction mixture. No organic solvent was added and the reaction products were identified by ^19^F NMR. Results are given in Table 2.

As we might expect, the results strongly depend on the catalyst used. Reactions without POM-IL do not work (entry A). The two polyoxomolybdates, namely **3** and **4**, clearly display less oxidizing power since only 50% and 33% conversion of the alcohol are measured in 2 h, respectively. Interestingly, compound **3**, (P_6,6,6,14_)_4_[PMo_11_VO_40_], shows no selectivity and poor efficiency, while the use of material **4**, (P_6,6,6,14_)_6_[P_2_Mo_18_O_62_], despite low conversion, seems to be selective and the major product obtained is the corresponding aldehyde (entries D and E).

On the other hand, the two polyoxotungstates catalysts—compounds **1** and **2**—appear more efficient for this oxidation reaction with an almost quantitative conversion of the alcohol mainly into the corresponding carboxylic acid within 2 h. The yield in acid compound is indeed higher than 86% (entry B and C). Therefore, depending on the objectives of their uses, polyoxotungstates will be favored if strong oxidation is required, while polyoxomolybdates will favor soft but more selective catalytic reactions.

An important aspect in the development of eco-compatible catalytic processes concerns the recyclability and the reusability of the catalyst, as well as the isolation of the reaction product. The two polyoxotungstates compounds are selected for the following of this study. After reaction, the organic products were separated from POM-IL phase thanks to a reusable steric exclusion column. After addition of a minimum amount of THF, the reaction mixture was added allowing to separate the POM-IL from the organic molecules. With this methodology that we developed, the POM-ILs can be easily recovered.

Of course, to be reusable the catalyst must be intact after catalysis. After separation from the reaction mixture, the two POM-ILs ((P_6,6,6,14_)_4_[W_10_O_32_] (**1**) and (P_6,6,6,14_)_8_[ɣ-SiW_10_O_36_]) (**2**) were analyzed by FT-IR to evidence any degradation, which could be detrimental for a possible recycling. The FT-IR spectra are given in the supporting Information (Figures S14 and S15). They evidence that the compound **1** is preserved by such a treatment and can be reused. In contrast, the FT-IR spectrum of **2** after catalytic reaction is dramatically modified in the region of vibration bands typical of the POM. It means that divacant Keggin-type structure [ɣ-SiW_10_O_36_]^8−^ degrades during the process and therefore cannot be recycled.

To complete this study, we focused our attention only on the compound **1**, which appears as the most promising. As a proof of concept, the reactivity of **1** with a small series of organic alcohols was investigated in similar conditions, with a reaction time fixed at 16 h instead of 2 h to have complete conversion. In all cases, the major oxidized products were identified as carboxylic acids. The results are given in Table 3.

The process works efficiently on benzylic alcohols as acids are obtained in good yields (entries 1 and 2). However, when they have a nitro group in the *para* position, the yield drops to around 60% (entry 3). This drop is even more marked when the benzyl alcohol is in position 2 of a pyridine (entry 6). For aliphatic primary alcohols, the isolated yield is correct. The 3-phenylpropan-1-ol gives a 63% yield (entry 4). This can be explained by the presence of many products; unfortunately, they could not be separated, clearly identified and quantified due to the complexity of the mixtures formed. This is even more the case for the oxidation of its secondary alcohol analogue to a ketone as only 32% of the yield was isolated pure (entry 7). The average yield obtained with fatty alcohol hexadecanol is mainly due to solubility problems in POM-IL, nevertheless the yield is still rather satisfactory (entry 5). Further optimizations of the reaction conditions and more complex systems are currently under development in our laboratory.

## 3. Materials and Methods

### 3.1. General Methods

Fourier Transform Infrared (FT-IR) spectra were recorded on a 6700 FT-IR Nicolet spectrophotometer (Thermo Fisher Scientific, Waltham, MA, USA), using the diamond ATR technique. The spectra were recorded on non-diluted compounds and ATR correction was applied. Thermogravimetric Analyses (TGA) were recorded on a TG/DTA 320 thermogravimetric balance (Seiko, Tokyo, Japan). The samples were measured between room temperature and 700 °C (scan rate: 5 °C min^−1^, under O_2_). Differential scanning calorimetry (DSC) was performed on a DSC 200 F3 instrument (NETZSC, Selb, Germany) equipped with an N_2_ cooler, allowing measurements from −170 °C up to 450 °C. The samples were examined at a scanning rate of 10 K.min^−1^ by applying two heating and one cooling cycles. The apparatus was calibrated with indium (156.6 °C). ^1^H-(300 MHz) NMR, ^31^P-(121.5 MHz) NMR and ^19^F-(188 MHz) NMR spectra were recorded at room temperature on an AC-300 spectrometer (Bruker, Billerica, Massachusetts, USA) in (CD_3_)_2_CO, CDCl_3_ and (CD_3_)_2_SO. Chemical shifts are reported in parts per million (ppm) relative to internal references. The residual peaks of (CD_3_)_2_CO (2.05 ppm), CDCl_3_ (7.26 ppm) or (CD_3_)_2_SO (2.5 ppm) for ^1^H (300 MHz) NMR spectra and CFCl_3_ (0.00 ppm) as the internal reference for ^19^F-NMR spectra. Liquid ^183^W (16.7 MHz) and ^29^Si (56.3 MHz) NMR spectra were obtained on a high resolution 400 MHz Bruker Avance spectrometer, equipped with 10 mm BBO probes or BBI 5 mm probes with Z gradient, respectively. CD_3_CN or (CD_3_)_2_CO were used as a solvent. Polarized optical microscopy (POM) investigations were performed on a H600L polarizing microscope (Nikon, Shinjuku, Japan) equipped with a LTS420 “liquid crystal pro system” hot-stage (Linkam, Tadworth, UK). Rheological measurements were performed on a Haake MARS III (Thermo Fisher Scientific, Waltham, MA, USA) controlled-stress rheometer equipped with a cone-plate geometry (diameter = 35 mm, angle = 1°) and a Peltier thermal regulator.

### 3.2. Syntheses

#### 3.2.1. Chemicals

All reagents were purchased from commercial sources and used without further purification. The POMs (K_8_[ɣ-SiW_10_O_36_]·12H_2_O, Na_6_[P_2_Mo_18_O_62_]·20H_2_O and H_4_[PMo_11_VO_40_]·32.5H_2_O) were synthetized as described in the literature. [72,73,74] Styrene divinylbenzene beads S-X1 for size exclusion chromatography, 1% crosslinkage, 40–80 µm bead size, 600–14,000 MW exclusion range provided by Bio-Rad (Hercules, California, USA) was used to recycle POM-ILs.

#### 3.2.2. Synthesis of POM-Based Ionic Liquids: Synthesis of (P_6,6,6,14_)_4_[W_10_O_32_] (**1**)

This synthesis was performed as described in the literature [72]. In a 250 mL beaker flask, sodium tungstate dihydrate (16 g, 50 mmol) was dissolved in 100 mL of boiling distilled water. Then, 33.5 mL of boiling HCl (3 M) was added with a rapid stirring. After 2 min of strong boiling, tetradecyltrihexylphosphonium chloride (P_6,6,6,14_Cl) (7.55 g, 15 mmol) in 10 mL of ethanol was added. The polyoxometalate-based ionic liquid formed a dense phase in the bottom of the beaker. Finally, the aqueous phase was separated, and the POM-IL phase was washed 3 times with 40 mL of boiling distilled water and dried with a vacuum pump until the POM-IL became a colorless viscous liquid. Yield 15 g, 72% based on tungstate. IR/cm^−1^: 2954 (s), 2926 (vs), 2855 (s), 1466 (m), 1408 (w), 1378 (w), 1212 (vw), 1112 (vw), 723 (m), 994 (vw), 958 (s), 891 (s), 805 (vs), 586 (w), 435 (m), 404 (m), 348 (vw), 335 (w). ^1^H-NMR (300 MHz, (CD_3_)_2_CO): δ (ppm) 2.48 (m, 8H), 1.71 (m, 8H), 1.56 (m, 8H), 1.4–1.2 (m, 34H) and 0.88 (m, 12H). ^31^P-NMR (121.5 MHz, (CD_3_)_2_CO): δ (ppm) 33.95. ^183^W-NMR (16.7 MHz, (CD_3_CN)): δ (ppm) −20.9 (s, 8W), −163 (s, 2W). TGA: A weight loss of 39.4% between RT and 700 °C corresponds to a combustion of 4 cations (P_6,6,6,14_)^+^. Thermogravimetric analyses show that the compound is stable up to 200 °C without any degradation and a total absence of water.

#### 3.2.3. General method for the synthesis of other POM-ILs (Compounds **2**, **3** and **4**)

The syntheses of POM-ILs were mainly prepared by exchanging protons or the alkali counter cations by organic ones. The simplest method consisted in mixing the POM dissolved in 10 mL of water with the organic salt dissolved in 10 mL of water or in a miscible organic solvent (often an alcohol) in the right stoichiometry. After 24 h of reaction under stirring, the two phases were separated. The heavy phase was the POM-IL which formed a new liquid phase. After several washings with water (3 × 10 mL), the resulting hybrid POM-IL was dried and analyzed.

*(P_6,6,6,14_)_8_[SiW_10_O_36_]·3.7P_6,6,6,14_Cl 10.5H_2_O* (**2**): A mixture of P_6,6,6,14_Cl (3.4 g, 6.4 mmol, 8.2 eq.) and K_8_[γ-SiW_10_O_36_]·12H_2_O (2.34 g, 0.79 mmol, 1 eq.) was used. Yield 4.47 g, 90%. ^1^H-NMR (200 MHz, CDCl_3_): δ (ppm) 2.52 (m, 8H), 1.74 (m, 8H), 1.58 (m, 8H), 1.35 (m, 16H), 1.3–1.2 (s, 20H) et 0.88 (t, 12H). ^31^P-NMR (121.5 MHz, CDCl_3_): δ (ppm) 34.3. ^29^Si NMR ((56.3 MHz, CD_3_CN): δ (ppm) −84.5. IR/cm^−1^: 2954 (s), 2925 (vs), 2854 (vs), 1465 (m), 984 (w), 935 (w), 875 (m), 833 (w), 747 (m). TGA: A weight loss of 64.6% between RT and 700 °C corresponds to a combustion of 11.7 cations (P_6,6,6,14_)^+^. Moreover, a weight loss of 3% between RT and 150 °C corresponds to a loss of 10.5 H_2_O.

*(P_6,6,6,14_)_4_[PMo_11_VO_40_]* (**3**): A mixture of P_6,6,6,14_Cl (5.3 g, 1.2 mmol, 5.2 eq.) and H_4_[PMo_11_VO_40_]·32.5H_2_O (4.72 g, 1.96 mmol, 1 eq.) was used. Yield 5.2 g, 91%. ^1^H-NMR (200 MHz, CDCl_3_): δ (ppm) 2.38 (m, 8H), 1.56 (m 16H), 1.4–1.1 (m, 32H) et 0.88 (t, 12H). ^31^P-NMR (121.5 MHz, CDCl_3_): δ (ppm) 34.3, −3.2. IR/cm^−1^: 2953 (s), 2924 (vs), 2853 (vs), 1457 (m), 1406 (w), 1372 (w), 1073 (w), 1053 (m), 942 (s), 867 (m), 795 (s). TGA: A weight loss of 46.4% between RT and 700 °C corresponds to a combustion of 4.1 cations (P_6,6,6,14_)^+^ according to the expected formula.

*(P_6,6,6,14_)_6_[P_2_Mo_18_O_62_]·0.3(P_6,6,6,14_Cl)* (**4**): A mixture of P_6,6,6,14_Cl (3.38 g, 6.5 mmol, 6.1 eq.) and Na_6_[P_2_Mo_18_O_62_]·20H_2_O (3.5 g, 1.07 mmol, 1 eq.) was used. Yield 5.52 g, 91%. ^1^H-NMR (200 MHz, CDCl_3_): δ (ppm) 2.52 (m, 8H), 1.74 (m, 8H), 1.58 (m, 8H), 1.35 (m, 16H), 1.3–1.2 (s, 20H) et 0.88 (t, 12H). ^31^P-NMR (121.5 MHz, CDCl_3_): δ (ppm) 34.3, −2.4. IR/cm^−1^: 2954 (s), 2926 (vs), 2854 (vs), 1461 (m), 1406 (vw), 1377 (vw), 1077 (s), 1000 (w), 935 (s), 904 (s), 877 (m), 837 (vs), 788 (vs). TGA: A weight loss of 46.9% between RT and 700 °C corresponds to a combustion of 6.3 cations (P_6,6,6,14_)^+^.

### 3.3. Experimental Procedure for Catalysis Experiments

#### 3.3.1. Experimental Procedures for the Oxidation of the 2-(Trifluoromethyl)benzyl Alcohol with Different POM-ILs

In a 100 mL flask equipped with condenser, 1 eq. of 2-(trifluoromethyl)benzyl alcohol and 0.1 eq. of catalyst were introduced. The mixture was stirred during a few minutes at 90 °C and then 50 eq. of hydrogen peroxide (30 wt% in water) was quickly added. This biphasic system was then heated at 90 °C during 2 h. The biphasic mixture was then cooled at room temperature and homogenized by adding 20 mL of acetone. A sample was then collected and a ^19^F NMR spectrum was recorded.


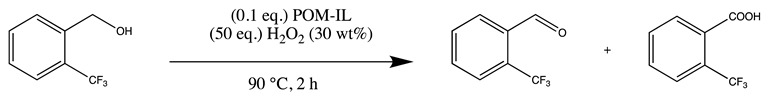


#### 3.3.2. Experimental Procedure for Oxidation of Alcohols

The experimental procedure used for catalysis is depicted below.


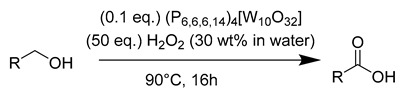


The following protocol was used for this study: In a 100 mL flask equipped with condenser, 1 eq. of alcohol and 0.1 eq. of catalyst ((P_6,6,6,14_)_4_[W_10_O_32_]) were introduced. The mixture was stirred during a few minutes at 90 °C and then 50 eq. of hydrogen peroxide (30 wt% in water) was quickly added. This biphasic system was kept at 90 °C for 16 h. The biphasic mixture was then cooled at room temperature and separated by decantation after adding 20 mL of distilled water. The aqueous phase was extracted 3 times with 20 mL of diethyl ether and the organic phase obtained was dried over MgSO_4_ and concentrated under reduced pressure. Considering the ionic liquid phase, acetone was added until a homogenous phase was obtained (around 20 mL). The solution was dried over MgSO_4_ and concentrated under reduced pressure. These two collected organic phases were mixed, and a minimum of tetrahydrofuran was added until a clear solution was obtained. The catalyst was then separated from the reaction products by a reusable steric exclusion polymer column composed of poly(styrene-co-divinylbenzene) eluted by tetrahydrofuran (around 100 mL). The POM-IL was the first to be recovered and, after the evaporation of THF, the catalyst was directly reused in another cycle. The oxidation products were collected right after the POM-IL from the column. When necessary, purification of reaction products was performed by recrystallization, pentane washing or silica plate. All data of the synthetized products are in accordance with those of compounds commercially available from Aldrich (Saint-Louis, Missouri, USA) and their analytical documents (see Aldrich website: https://www.sigmaaldrich.com).

## 4. Conclusions

As a conclusion of this work, we synthetized four POM-based ionic liquids, which were fully characterized by different techniques. Rheological investigations allowed us to demonstrate the true ionic liquid nature of these compounds. Nevertheless, we demonstrated that playing on the size and the charge of the POM one can dramatically modify the physical properties of the ionic liquid phase, especially in terms of viscosity and behavior. These variations are not well understood at the moment and further experimental work coupled with DFT calculation are needed to highlight the key parameters which govern the properties of such hybrid materials, notably the nature and the force of the interaction between POMs and organic cations.

These POM-ILs are able to dissolve organic molecules and therefore to act as solvent as do usual ionic liquids, but also as catalyst due to the POM when H_2_O_2_ is added as co-oxidant. We evidenced in this study that the POM-IL phase can be easily separated from reaction products and recycled for further uses by means of a steric exclusion gel. Besides, we demonstrated that the behavior of our four POM-ILs for catalysis is totally different in terms of power, selectivity and stability. The compound (P_6,6,6,14_)_4_[W_10_O_32_] appears as a very promising material and this study paves the way towards the use of such a POM-IL to develop sustainable processes of valorization of biomass, often constituted by recalcitrant biopolymers, for the production of biosourced valuable molecules.

## Figures and Tables

**Table 1:** Summary of the rheological properties of all the compounds investigated.

Compound	Glass Transition Temperature (°C)	Viscosity at 20 °C in Pa/s	Viscosity at 100 °C in Pa/s	Rheological Behavior	Class
P_66614_Cl	−64	0.86	0.04	Newtonian	liquid
(P_6,6,6,14_)_4_[W_10_O_32_]; **1**	−10	2200	0.92	Newtonian	liquid
(P_6,6,6,14_)_8_[SiW_10_O_36_]·3.7P_6,6,6,14_Cl 10.5H_2_O; **2**	−68	21	0.07	Dilatant	liquid
(P_6,6,6,14_)_4_[PMo_11_VO_40_]; **3**	−3.5	12,000	10.5	pseudoplastic	resin
(P_6,6,6,14_)_6_[P_2_Mo_18_O_62_]·0.3(P_6,6,6,14_Cl); **4**	−1	19,500	19	pseudoplastic	resin

**Table 2:**
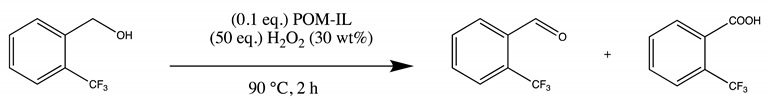
Oxidation of 2-trifluorobenzyl alcohol with POM-ILs as solvent and catalyst.

Entry ^a^	Catalyst	Conversion (%) ^b^	Aldehyde Yield (%) ^b^	Acid Yield (%) ^b^
A	/	3	0	0
B	**1**	98	6	86 (82) ^c^
C	**2**	96	0	90
D	**3**	50	21	21
E	**4**	33	26	0

^a^ Reaction conditions: 2-(trifluoromethyl)benzyl alcohol (1 eq.), H_2_O_2_ 30 wt% (50 eq.), POM-IL (0.1 eq.), 90 °C, 2 h. ^b^ Determined by ^19^F-NMR analysis using trifluoroanisole as internal standard. ^c^ Isolated yields after catalyst/product separation on a reusable steric exclusion polymer column.

**Table 3:** Scope of oxidation with (**1**), (P_6,6,6,14_)_4_[W_10_O_32_], as catalyst and H_2_O_2_ as co oxidant.

Entry ^a^	Substrate	Product	Yield (%) ^b^
1	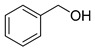	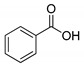	74
2	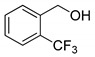	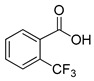	82
3	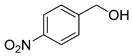	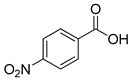	63
4	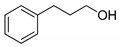	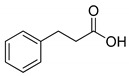	63
5	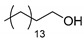	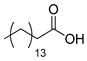	43
6	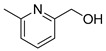	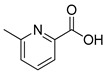	45
7	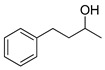	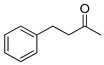	32

^a^ Reaction conditions: alcohol (1 eq.), H_2_O_2_ 30 wt% (50 eq.), (P_6,6,6,14_)_4_[W_10_O_32_] (0.1 eq.), 90 °C, 16 h. ^b^ Isolated yield after catalyst/product separation by a reusable steric polymer column and purification if necessary.

## Data Availability

The data presented in this study are available on request from the corresponding authors.

## Supplementary Materials

Electronic Supplementary Information (ESI) available: FT-IR spectra of starting salts of cations compared to those of POM-based materials (Figures S1–S4); ^183^W-, ^29^Si- and ^31^P-NMR spectra of compounds **1**–**4** (Figures S5–S8); ^1^H-NMR spectra of compounds **1**–**4** in CDCl_3_ or in acetone-d_6_ (Figures S9 and S10); DSC traces of P_6,6,6,14_Cl and compounds **1**, **3** and **4** (Figure S11), Pictures of P_6,6,6,14_Cl and compounds **1**, **3** and **4** under polarized optical microscope at various temperature (Figure S12); Rheological properties of P_6,6,6,14_Cl (Figure S13); FT-IR of compounds **1** and **2** before and after catalytic reaction (Figures S14 and S15).

## Sample Availability

Samples of the compounds presented in this study are available on request from the corresponding authors.
