# Supplementary material for: Synthesis, Physical Properties and Application of a Series of New Polyoxometalate-Based Ionic Liquids
Source: Molecules. 2021 Jan 18;26(2):496. doi: 10.3390/molecules26020496 (PMC7831901; doi:10.3390/molecules26020496)
Supplement: Supplementary file 1 [file molecules-26-00496-s001.pdf]

Synthesis, physical properties and application of a series of new polyoxometalate-based ionic liquids.

Yohan Martinetto,[1] Salomé Basset,[1] Bruce Pégot,[1] Catherine Roch-Marchal,[1] Franck Camerel,[2,*] Jelena Jeftic,[2] Betty Cottyn-Boitte,[3] Emmanuel Magnier,[1] and Sébastien Floquet,[1,*]

[1] Institut Lavoisier de Versailles, UMR 8180 CNRS, Université de Versailles St-Quentin en Yvelines, Université Paris-Saclay, 78035 Versailles, France.

[2] Institut des sciences chimiques de Rennes, UMR 6226, Université de Rennes 1, 35042 Rennes, France.

[3] Institut Jean-Pierre Bourgin, INRA, Agro Paris Tech, Université Paris Saclay, 78000 Versailles, France.

Corresponding authors: sebastien.floquet@uvsq.fr, franck.camerel@univ-rennes1.fr

Supporting Information

Characterizations by FT-IR spectra

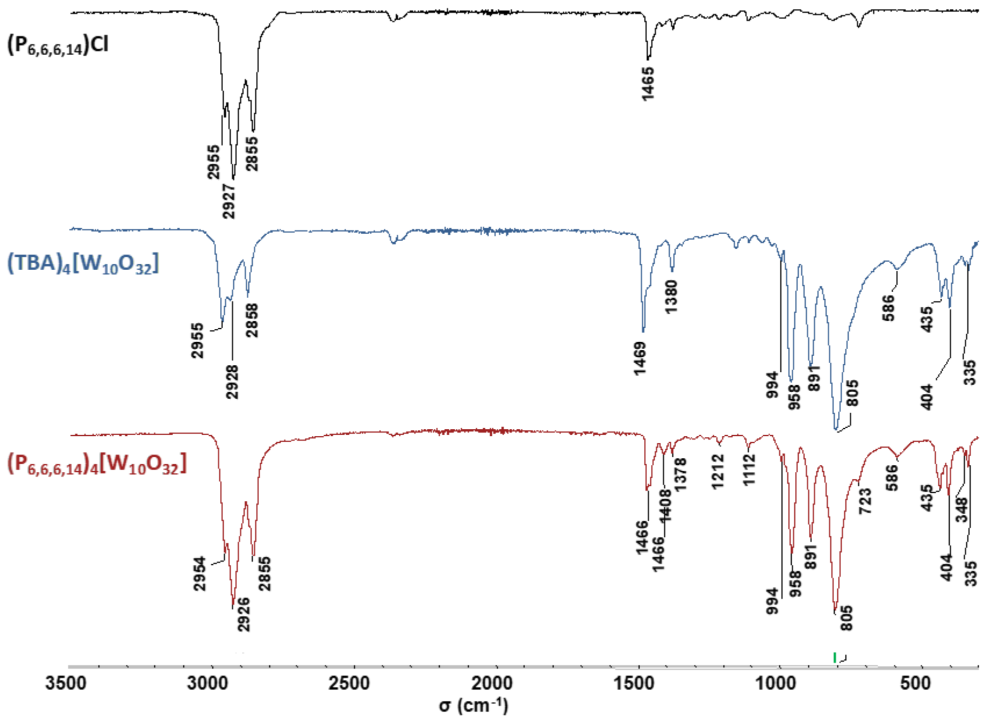

Figure S1: FT-infrared spectra comparing $(P_{6,6,6,14})_4[W_{10}O_{32}]$ (**1**) with $(TBA)_4[W_{10}O_{32}]$ and $P_{6,6,6,14}Cl$ as references.

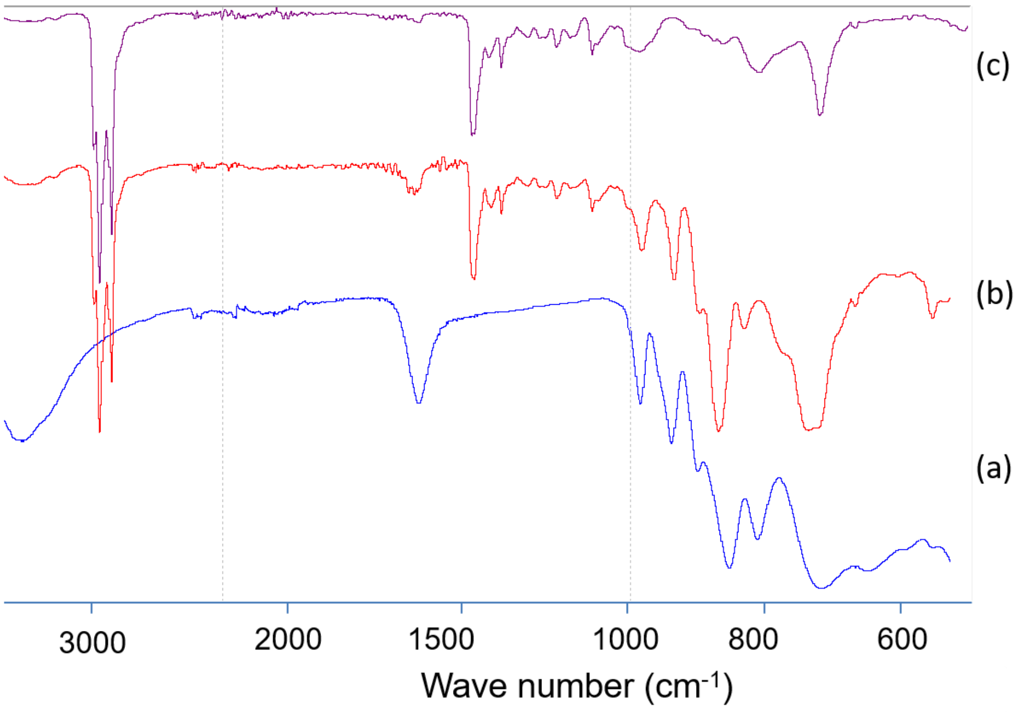

Figure S2: Infrared spectra comparing $(P_{6,6,6,14})_8[SiW_{10}O_{36}]\cdot 3.7P_{6,6,6,14},Cl\ 10.5H_2O$ (**2)** (b) with $P_{6,6,6,14}Cl$ (c) and the POM precursor $K_8[SiW_{10}O_{36}]$ (a)

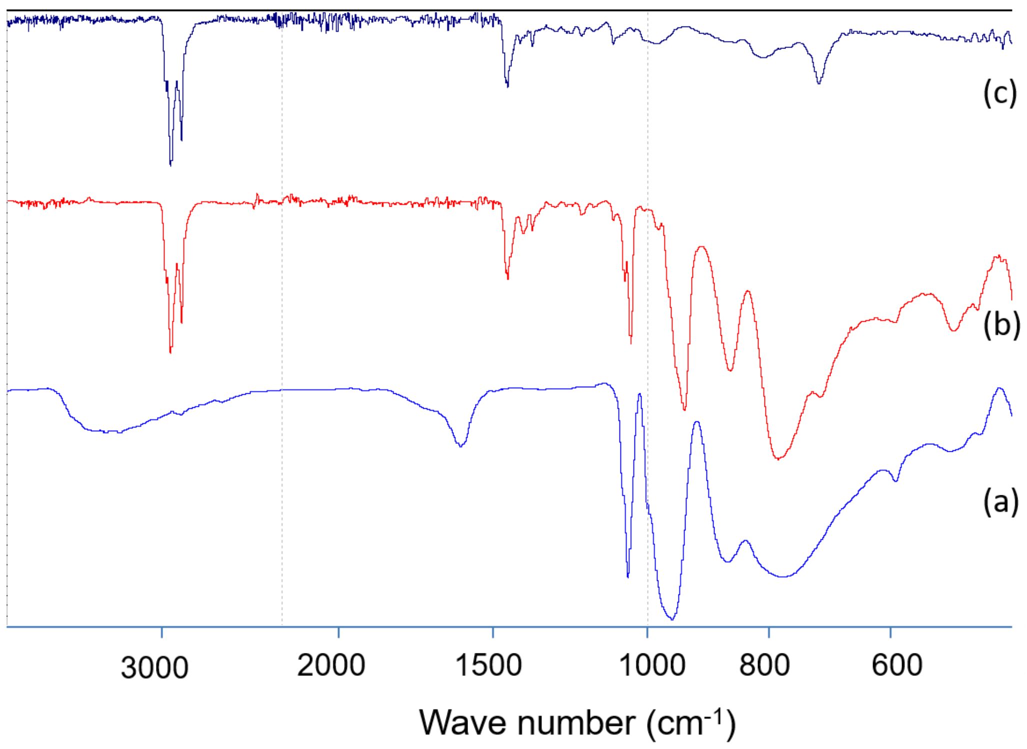

Figure S3: Infrared spectra comparing $(P_{6,6,6,14})_4PMo_{11}VO_{40}$(**3)** (b) with $P_{6,6,6,14}Cl$ (c) and the POM precursor $H_4PMo_{11}VO_{40}$ (a).

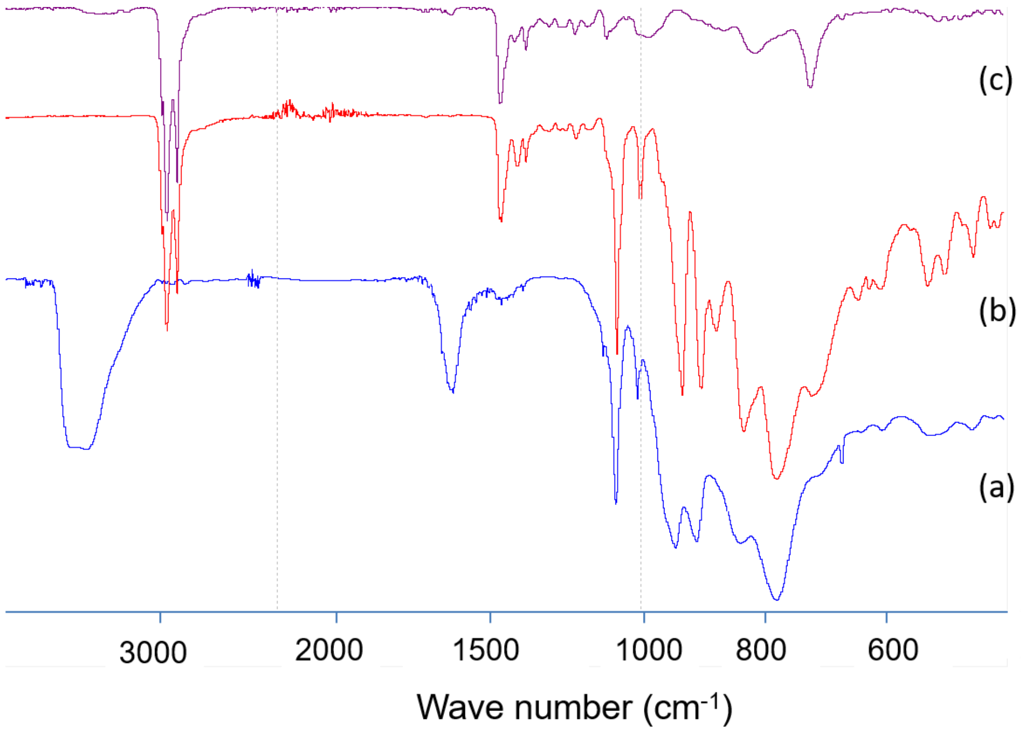

Figure S4: Infrared spectra comparing $(P_{6,6,6,14})_6[P_2Mo_{18}O_{62}]\cdot 0.3P_{6,6,6,14}Cl$ (**4)** (b) with $P_{6,6,6,14}Cl$ (c) and the POM precursor $Na_6P_2Mo_{18}O_{62}$ (a)

Characterizations by NMR in solution

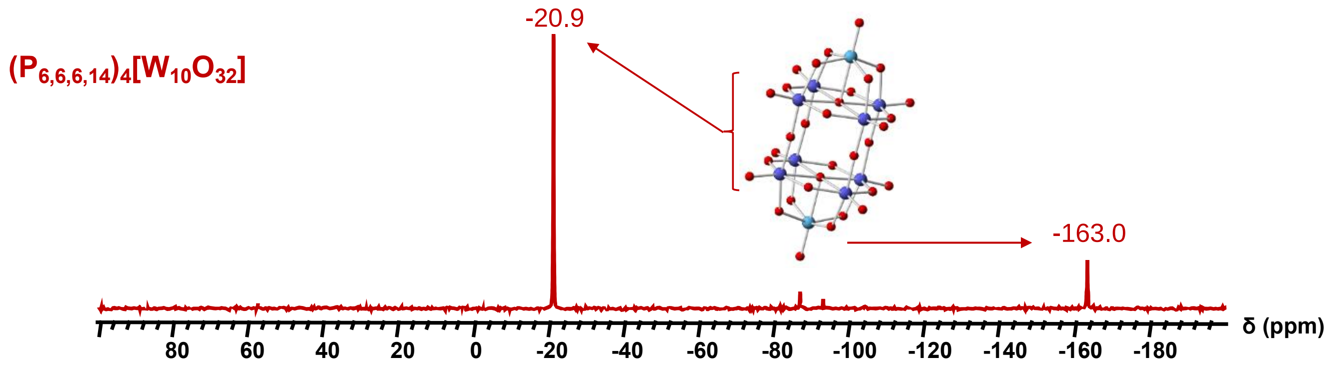

Figure S5: ^{183}W NMR spectrum of $(P_{6,6,6,14})_4[W_{10}O_{32}]$ (**1**) in CD_3CN

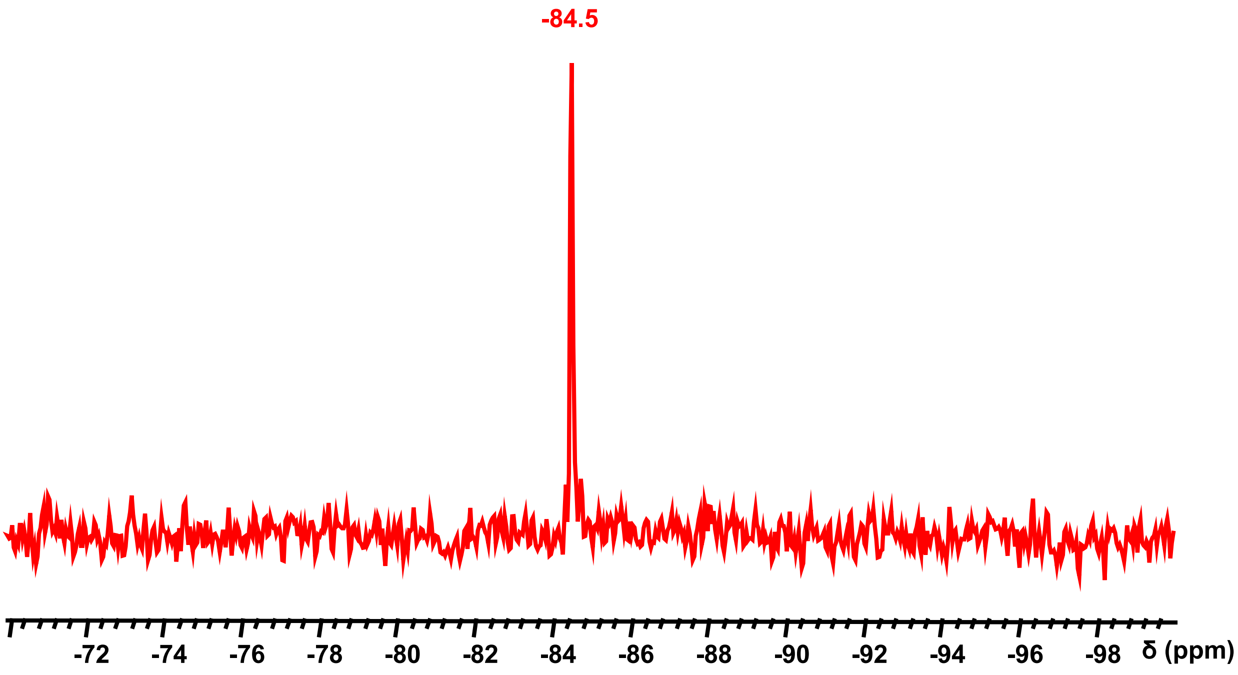

Figure S6: ^{29}Si NMR spectrum of $(P_{6,6,6,14})_8[SiW_{10}O_{36}]$ (**2**) in CD_3CN

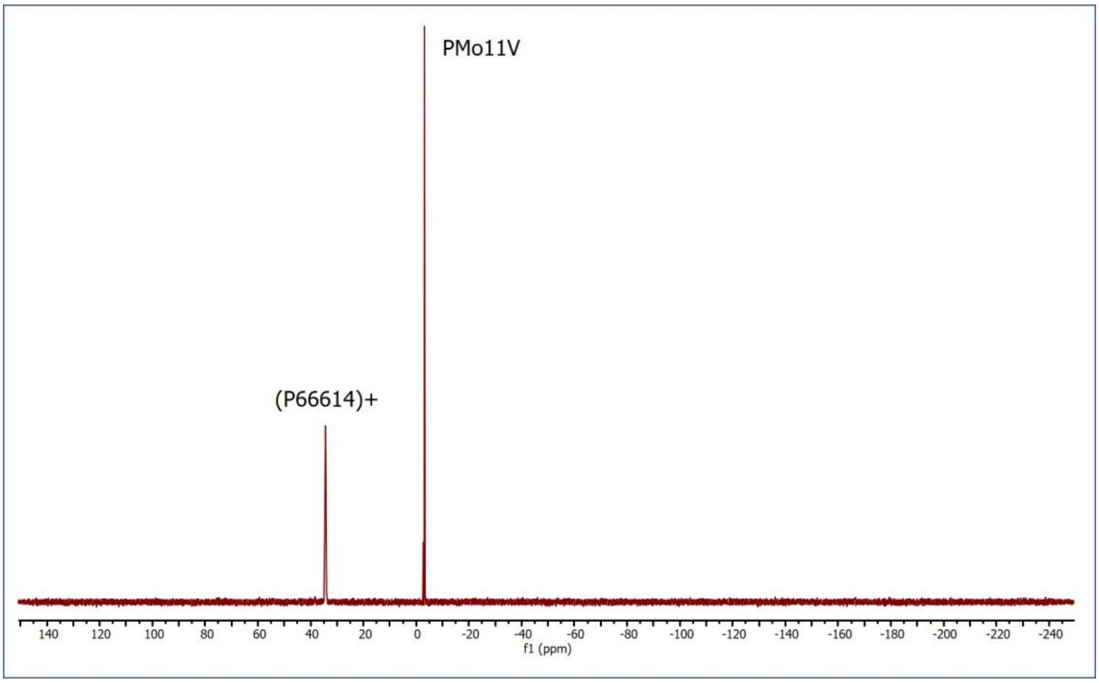

Figure S7: ^{31}P NMR spectrum of $(P_{6,6,6,14})_4[PMo_{11}VO_{40}]$ (**3**) in acetone.

*Note that due to different values of relaxation time of phosphorus atom in POM and in the cation, the integration ratio is not respected.

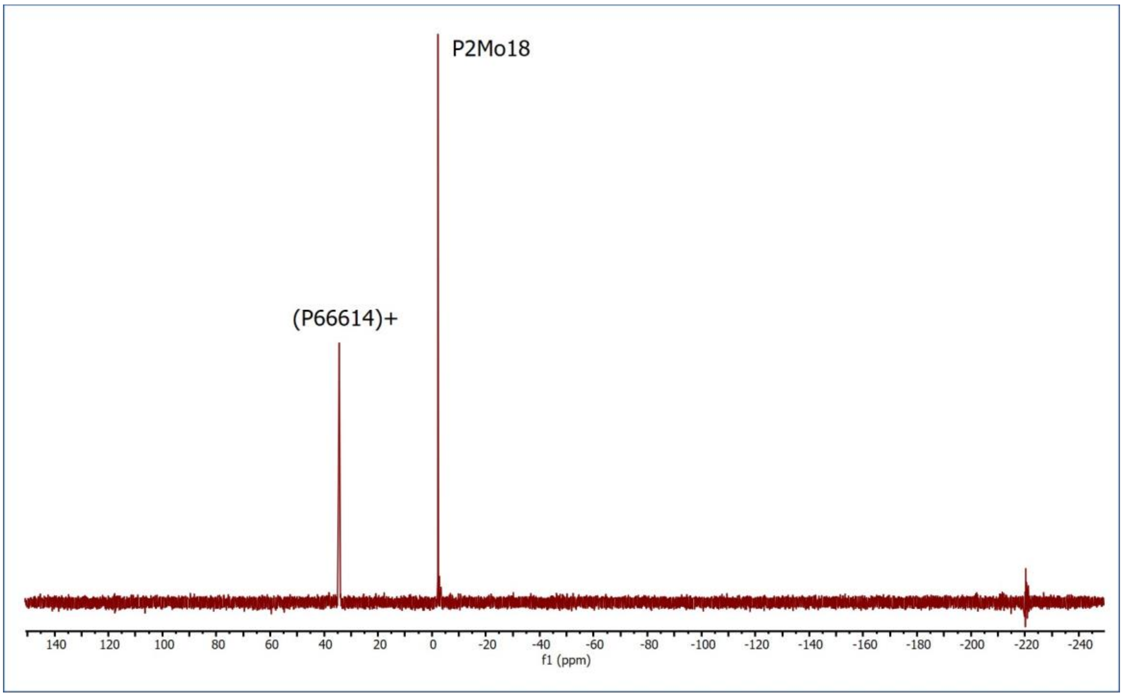

Figure S8: ^{31}P NMR spectrum of $(P_{6,6,6,14})_6[P_2Mo_{18}O_{62}]$ (**4**) in acetone.

*Note that due to different values of relaxation time of phosphorus atom in POM and in the cation, the integration ratio is not respected.

^{1}H NMR spectra.

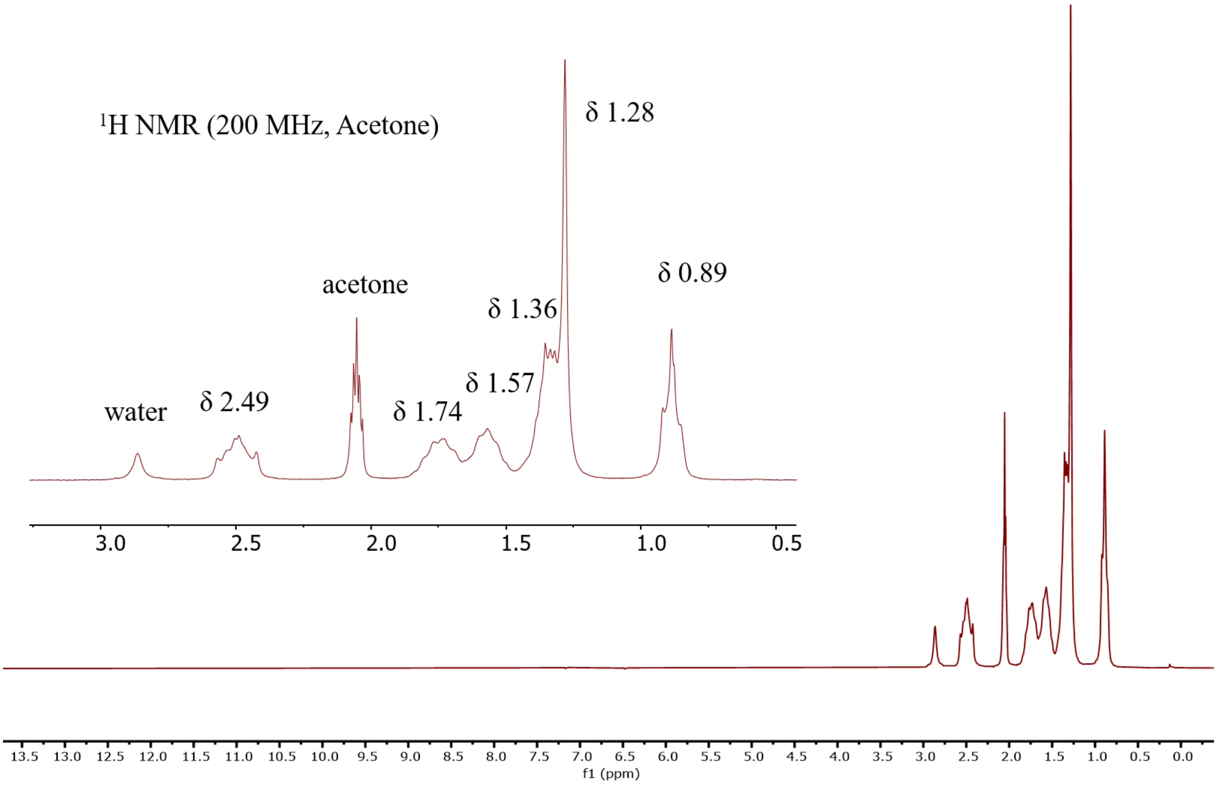

Figure S9: ^{1}H NMR spectrum of $(P_{6,6,6,14})_4[PMo_{11}VO_{40}]$ (**3**) in acetone.

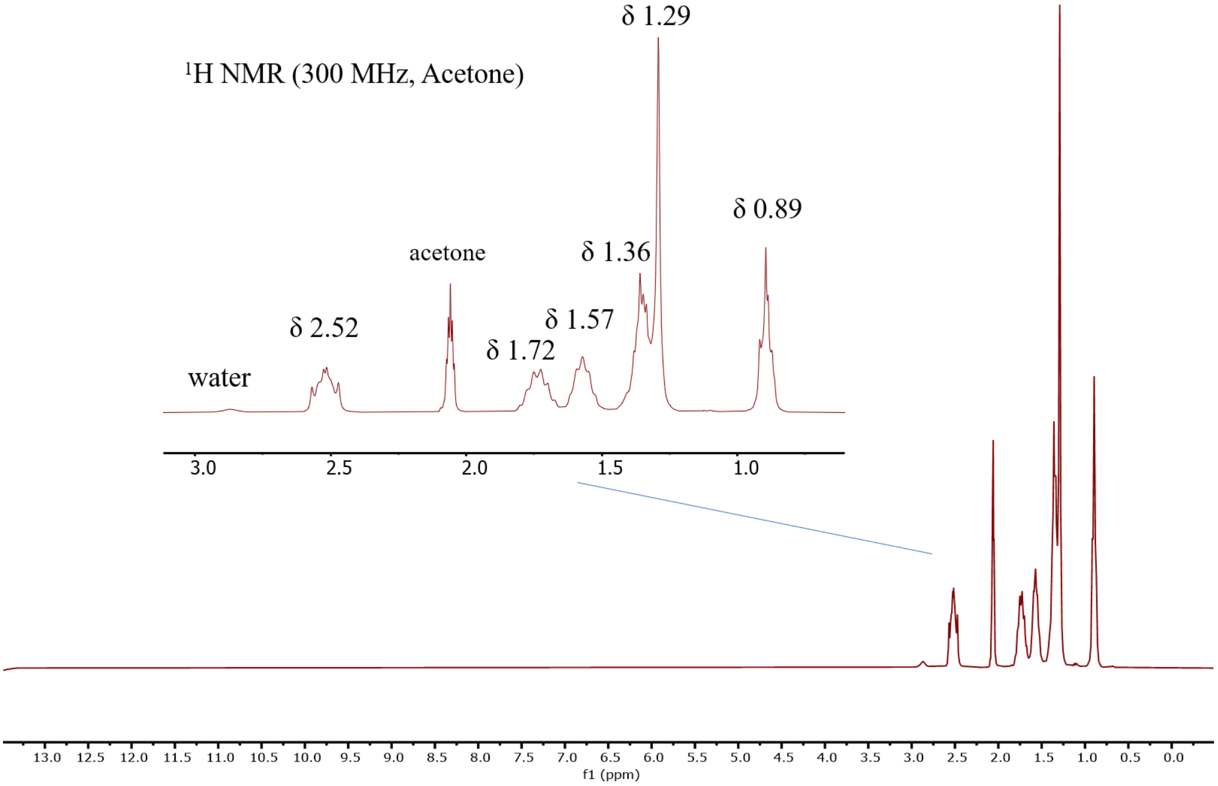

Figure S10: ^{1}H NMR spectrum of $(P_{6,6,6,14})_6[P_2Mo_{18}O_{62}]$ (**4**) in acetone.

Physical properties of POM-ILs

Differential Scanning Calorimetry

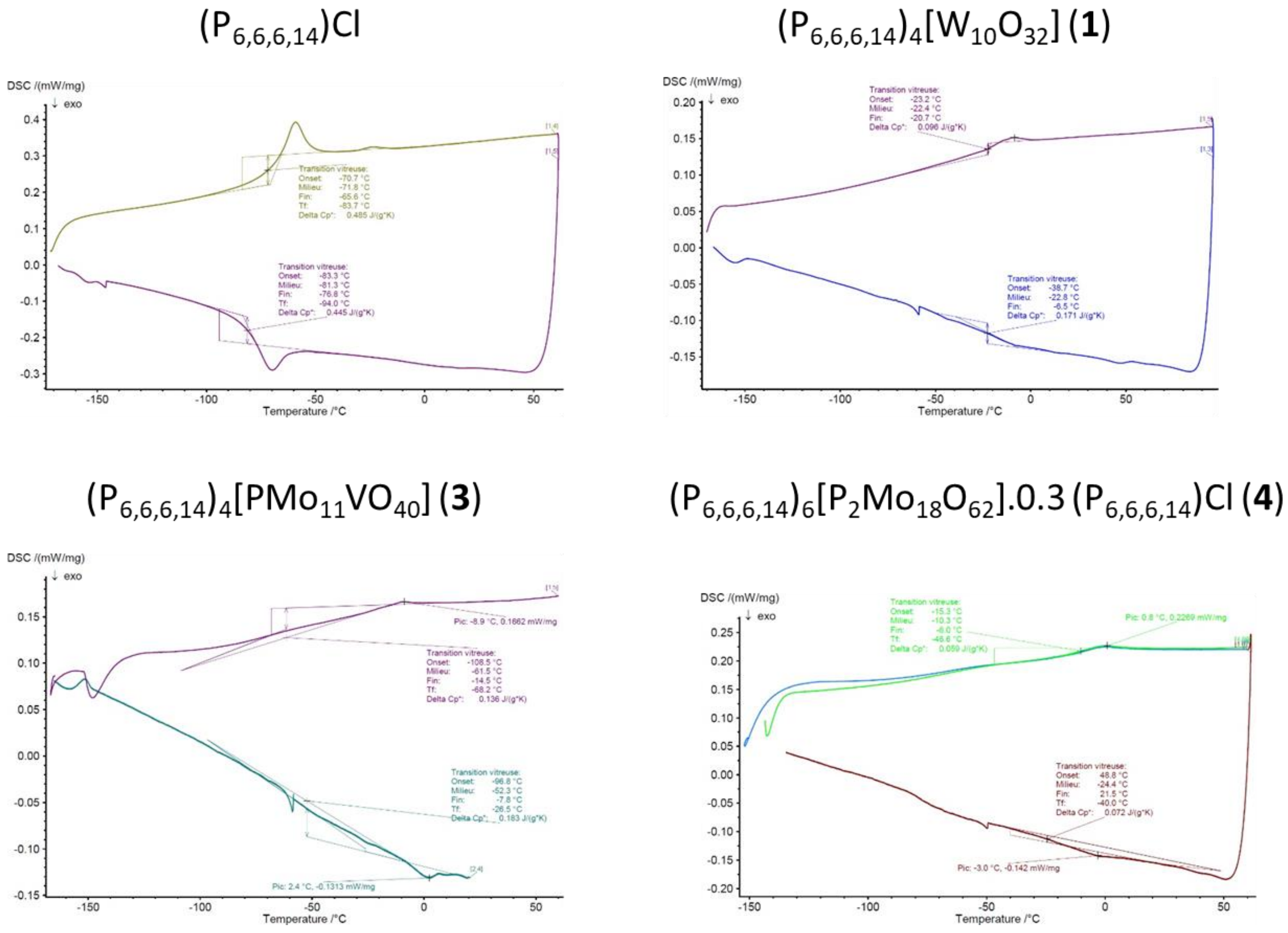

Figure S11: DSC Curves in heating and in cooling modes of compounds **1**, **3** and **4** in comparison with the salt $P_{6,6,6,14}Cl$.

Polarized Optical Microscopy

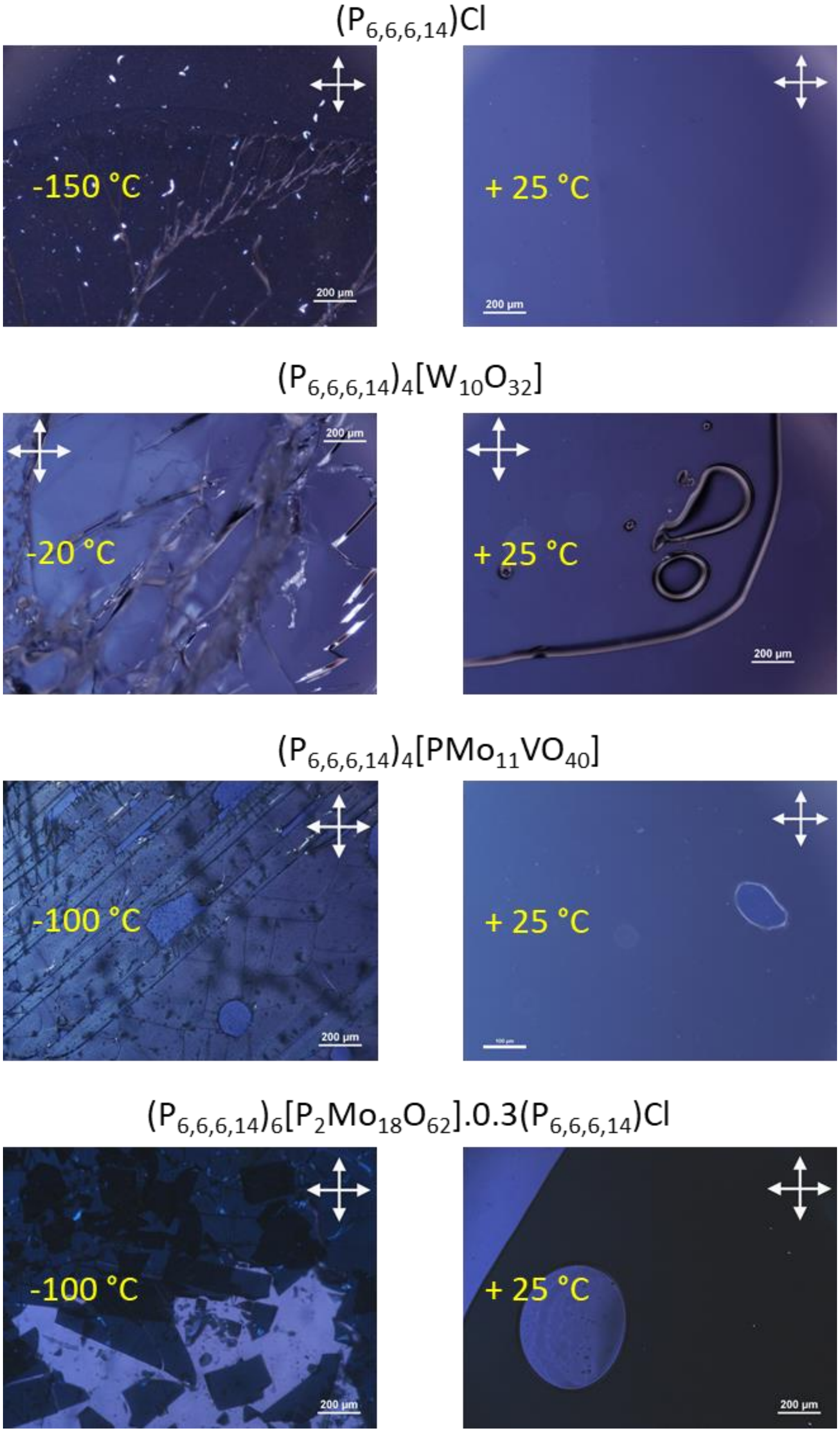

Figure S12: From top to the bottom : Optical photomicrograph of $P_{66614}Cl$ obtained with a polarizing microscope at -150 °C and at +25 °C (The liquid is on the right side of the picture); Optical photomicrograph of $(P_{6,6,6,14})_4[W_{10}O_{32}]$ (**1**) obtained with a polarizing microscope at +25 °C and at -20 °C; Optical photomicrograph of $(P_{66614})_4PMo_{11}VO_{40}]$ (**3**) obtained with a polarizing microscope at +25 °C after heating at 120 °C and in its solid state at -100 °C; Optical photomicrograph of $(P_{6,6,6,14})_6[P_2Mo_{18}O_{62}].0.3P_{6,6,6,14}Cl$ (**4**) obtained with a polarizing microscope at +25°C and at -100°C after heating at 120 °C.

Rheological properties

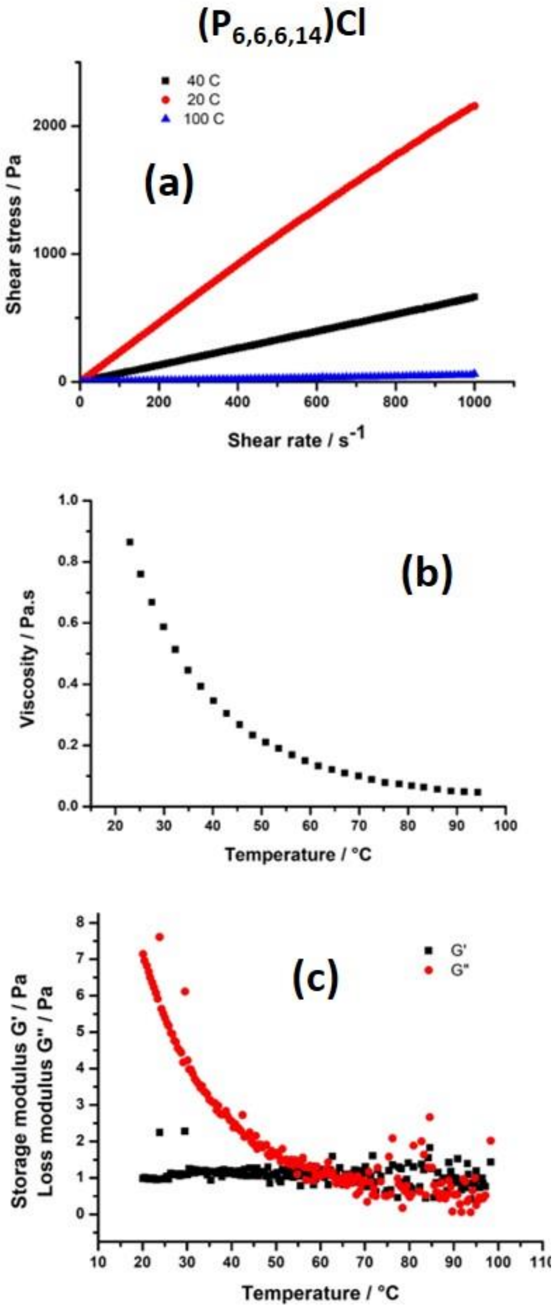

Figure S13: Flow curves $t = f(\dot{\gamma})$ showing the evolutions of the shear stress versus the shear rate at various temperatures for the salt $P_{6,6,6,14}Cl$ (a). Each line corresponds to a curve $t = f(\dot{\gamma})$ for a given temperature; Viscosity of $P_{6,6,6,14}Cl$ as a function of the temperature between 20 °C and 100°C ($(\gamma)^{\cdot} = 10\ s^{-1}$) (b); Temperature dependence of G′ and G′′ (20% strain, f = 1Hz) for $(P_{66614})Cl$ (c)

Stability of the catalyst after catalytic process

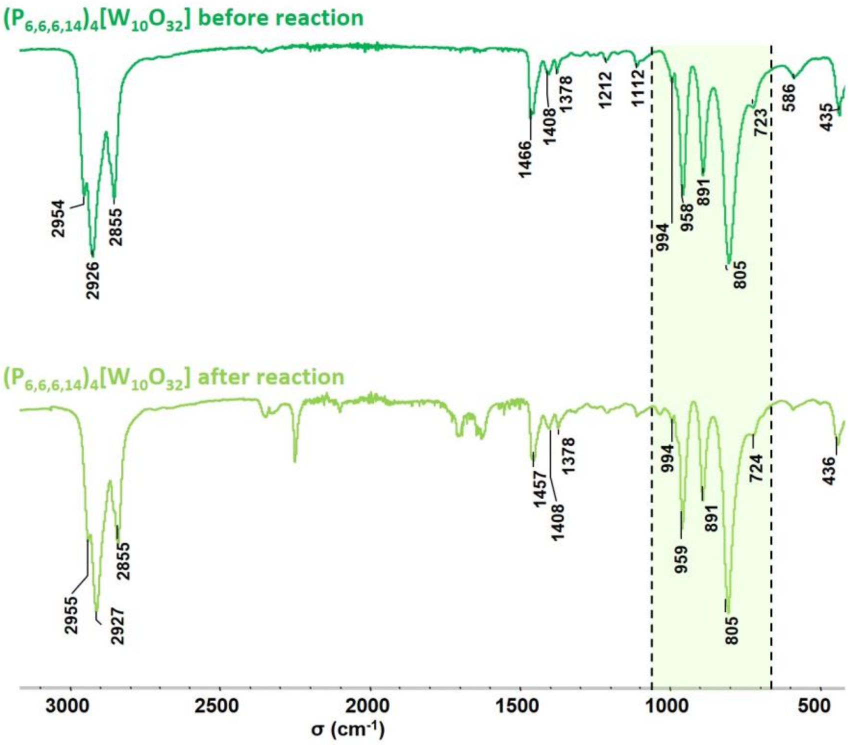

Figure S14: FT-IR spectra of $(P_{6,6,6,14})_4[W_{10}O_{32}]$ (**1**) recorded before and after catalytic process.

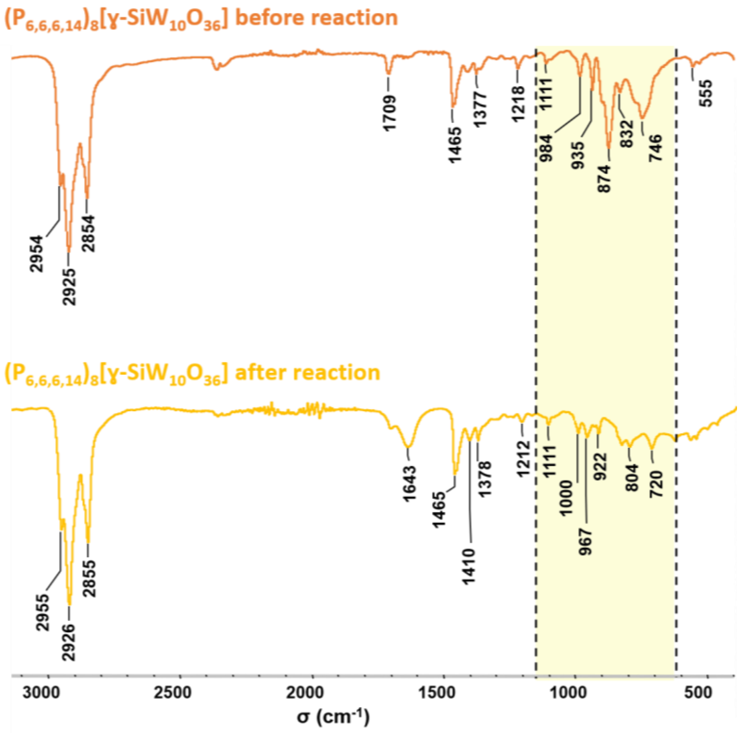

Figure S15: FT-IR spectra of $(P_{6,6,6,14})_8[\gamma\text{-}SiW_{10}O_{36}].3.7(P_{6,6,6,14})Cl$ (**2**) recorded before and after catalytic process.
